# The CLC-2 Chloride Channel Modulates ECM Synthesis, Differentiation, and Migration of Human Conjunctival Fibroblasts via the PI3K/Akt Signaling Pathway

**DOI:** 10.3390/ijms17060910

**Published:** 2016-06-09

**Authors:** Lixia Sun, Yaru Dong, Jing Zhao, Yuan Yin, Yajuan Zheng

**Affiliations:** 1 Department of Ophthalmology, Second Hospital of Jilin University, Jilin University, Changchun 130041, China; sunlx_2008@163.com (L.S.); dongyaru@sina.com (Y.D.); lhbswqw@126.com (J.Z.); chaizg2008@163.com (Y.Y.); 2 Department of Ophthalmology, China-Japan Union Hospital, Jilin University, Changchun 130031, China

**Keywords:** *CLC-2* siRNA, TGF-β1 differentiation, extracellular matrix, human conjunctival fibroblasts, migration, signaling pathway

## Abstract

Recent evidence suggests that chloride channels are critical for cell proliferation, migration, and differentiation. We examined the effects of transforming growth factor (TGF)-β1 on chloride channel expression and associations with human conjunctival fibroblast (HConF) biology. To investigate the potential role of chloride channel (CLC)-2 in migration, transition to myofibroblasts and extracellular matrix (ECM) synthesis of HconF, a small interfering RNA (siRNA) approach was applied. TGF-β1-induced migration and transition of fibroblasts to myofibroblasts characterized by α-smooth muscle actin (α-SMA) expression, supported by increased endogenous expression of CLC-2 protein and mRNA transcripts. ECM (collagen I and fibronectin) synthesis in HConF was enhanced by TGF-β1. *CLC-2* siRNA treatment reduced TGF-β1-induced cell migration, transition of fibroblasts to myofibroblasts, and ECM synthesis of HConF. *CLC-2* siRNA treatment in the presence of TGF-β1 inhibited phosphorylation of PI3K and Akt in HConF. These findings demonstrate that CLC-2 chloride channels are important for TGF-β1-induced migration, differentiation, and ECM synthesis via PI3K/Akt signaling in HConF.

## 1. Introduction

Postoperative scarring is the most common reason for failure of glaucoma filtering surgery. At present, anti-metabolic drugs are used to prevent subconjunctival scar formation in high-risk patients, but severe complications associated with the use of these agents limit their application [1]. Therefore, identification of methods of reducing subconjunctival scar formation is of utmost significance.

Transformation of fibroblasts into myofibroblasts, migration of fibroblasts, and excessive deposition of extracellular matrix (ECM) are important steps of scar formation [2,3]. Transforming growth factor β (TGF-β) is recognized as a major driver of postoperative scarring after glaucoma surgery [4,5], that increases collagen deposition, inhibits collagen degradation, and promotes transformation of fibroblast into myofibroblasts and migration of myofibroblasts [6,7]. Approaches that inhibit differentiation, migration and ECM synthesis of fibroblasts can alleviate the postoperative scarring after glaucoma surgery.

Chloride channels are expressed in nearly all eukaryotic cells. Volume-activated chloride channels (VACCs) participate in cell proliferation and apoptosis through cell swelling and shrinkage, respectively [8,9,10]. Studies have also demonstrated that chloride channels are involved in migratory capacity and cell proliferation [11,12]. Chloride channe (CLC)-3 overexpression was previously shown to promote fibroblast-to-myofibroblast transition reflected by increased α-smooth muscle actin (α-SMA) protein expression, and *ClC-3* knockdown by siRNA inhibited the fibroblast-to-myofibroblast transition in the presence of TGF-β1 in human corneal keratocytes [13]. Fuqiang *et al.* [14] found that RNAi knockout of the *CLC-2* gene inhibited high glucose-induced migration of rat keratinocytes via inhibition of phosphatidyl-Inositol 3-kinase (PI3K) signaling. Together, these studies have provided direct evidence of the physiological relationship between chloride channels and fibroblast-to-myofibroblast transdifferentiation in addition to proliferation, migration, and ECM production in many cell types. However, studies on the role of chloride channels in human conjunctival fibroblasts after glaucoma filtering surgery are limited. The present study applied *CLC-2* knockdown in human conjunctival fibroblasts (HconF) to determine whether CLC-2 participates in the fibroblast-to-myofibroblast transition, migration of myofibroblasts, and ECM synthesis in the presence of TGF β1 to explore effective methods to prevent scarring in glaucoma filtering surgery.

## 2. Results

### 2.1. Effect of CLC-2 siRNA and TGF-β1 on CLC-2 Expression in Human Conjunctival Fibroblast (HConF)

**Figure 1:**
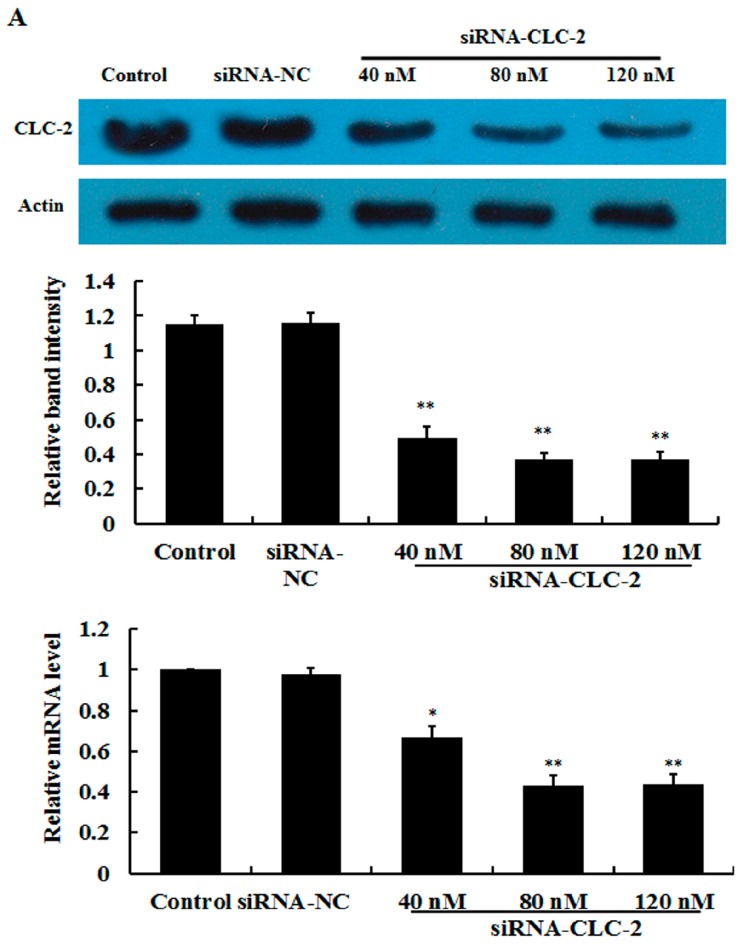

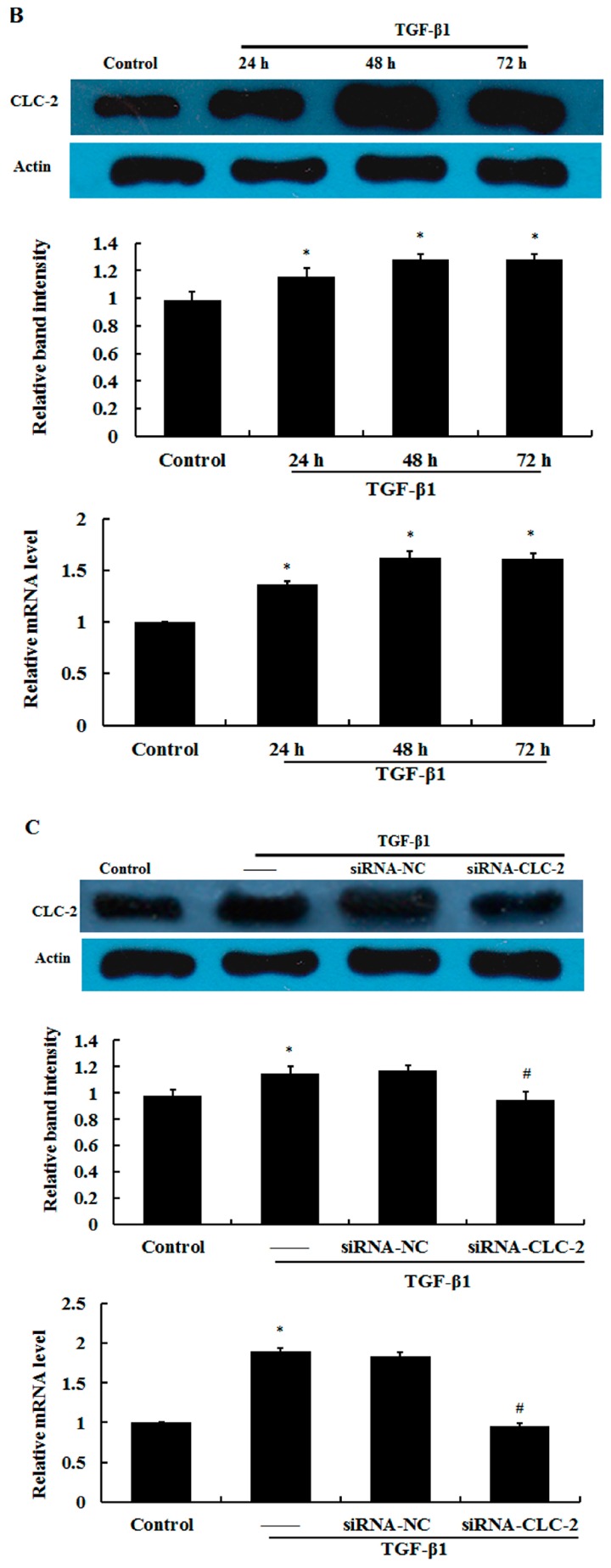
Effect of *CLC-2* siRNA and transforming growth factor (TGF)-β1 on CLC-2 protein and mRNA expression in Human Conjunctival Fibroblast (HConF). (**A**) Effect of 40–120 nM *CLC-2* siRNA on CLC-2 protein and mRNA expression in HConF determined by western blot and reverse transcription quantitative polymerase chain reaction; (**B**) Effect of 2 ng/mL TGF-β1 on levels of CLC-2 protein and mRNA expression at 24, 48, and 72 h in HconF; (**C**) HConF transfected with *CLC-2* siRNA in the presence of TGF-β1 showed decreased CLC-2 protein and mRNA levels compared to nontransfected and mutant *CLC-2* siRNA transfected cells, whereas nontransfected and mutant siRNA transfected cells had similar CLC-2 protein and mRNA levels, (data are presented as the mean ± SD (*n* = 4); siRNA-NC: mutant *CLC-2* siRNA; * *p* < 0.05, ** *p* < 0.01 *vs.* control; # *p* < 0.05 *vs.* TGF-β1 in ANOVA).

In this study, ConFs transfection efficiency was determined by uptake of siRNA-labeled Alexa 488 (Figure S1). After transfection, levels of CLC-2 protein and mRNA were determined by western blot and reverse transcription quantitative polymerase chain reaction (RT qPCR) analyses, respectively. As shown in Figure 1A, after 48 h of treatment with siRNA, CLC-2 protein and mRNA expression were inhibited in a concentration-dependent manner. In addition, no significant additional effect was observed at the highest concentration. TGF-β1 (2 ng/mL) increased the *CLC-2* expression of HConF in a time dependent-manner and no significant additional effect was observed at the longest treatment duration (Figure 1B). HConF transfected with *CLC-2* siRNA in the presence of TGF-β1 showed reduced *CLC-2* expression compared to that in TGF-β1-treated nontransfected cells and TGF-β1-treated negative siRNA control cells (Figure 1C). This finding demonstrates that *CLC-2* siRNA inhibits TGF-β1-induced *CLC-2* expression.

### 2.2. Effect of CLC-2 siRNA on Myofibroblast Differentiation

**Figure 2:**
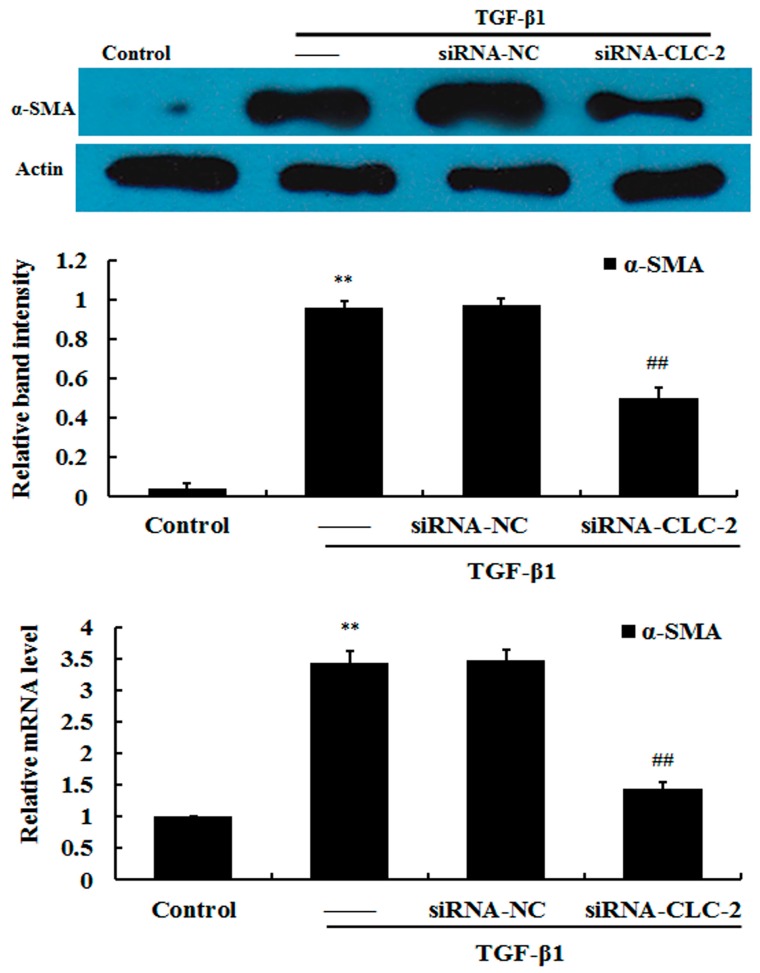
*CLC-2* knockdown attenuates transforming growth factor (TGF)-β1-induced α-smooth muscle actin (α-SMA) expression. Human conjunctival fibroblasts (HConF) treated with transforming growth factor (TGF)-β1 demonstrated elevated α-SMA mRNA and protein expression compared to untreated control cells, which exhibited minimal α-SMA expression. Cells transfected with *CLC-2* siRNA in the presence of TGF-β1 showed decreased α-SMA mRNA and protein expression levels compared to nontransfected and mutant *CLC-2* siRNA transfected cells, whereas nontransfected and mutant siRNA transfected cells had similar α-SMA expression levels; Data are presented as the mean ± SD (*n* = 4); siRNA-NC: mutant *CLC-2* siRNA; ** *p* < 0.01 *vs.* control; ## *p* < 0.01 *vs.* TGF-β1 in ANOVA.

The transition from fibroblasts-to-myofibroblasts is characterized by an increase in α-SMA expression in myofibroblasts. HConF treated with 2 ng/mL TGF-β1 displayed elevated α-SMA protein and mRNA expression compared to untreated control cells, which showed minimal α-SMA expression (Figure 2). This result demonstrates TGF-β1-induced myofibroblast differentiation. HConF transfected with *CLC-2* siRNA in the presence of TGF-β1 showed a 47.02% reduction in α-SMA protein expression compared to TGF-β1-treated nontransfected cells and TGF-β1-treated negative siRNA control cells. This finding demonstrates that *CLC-2* siRNA inhibits TGF-β1-induced myofibroblast differentiation.

### 2.3. Effect of CLC-2 siRNA on Extracellular Matrix Synthesis of HConF

**Figure 3:**
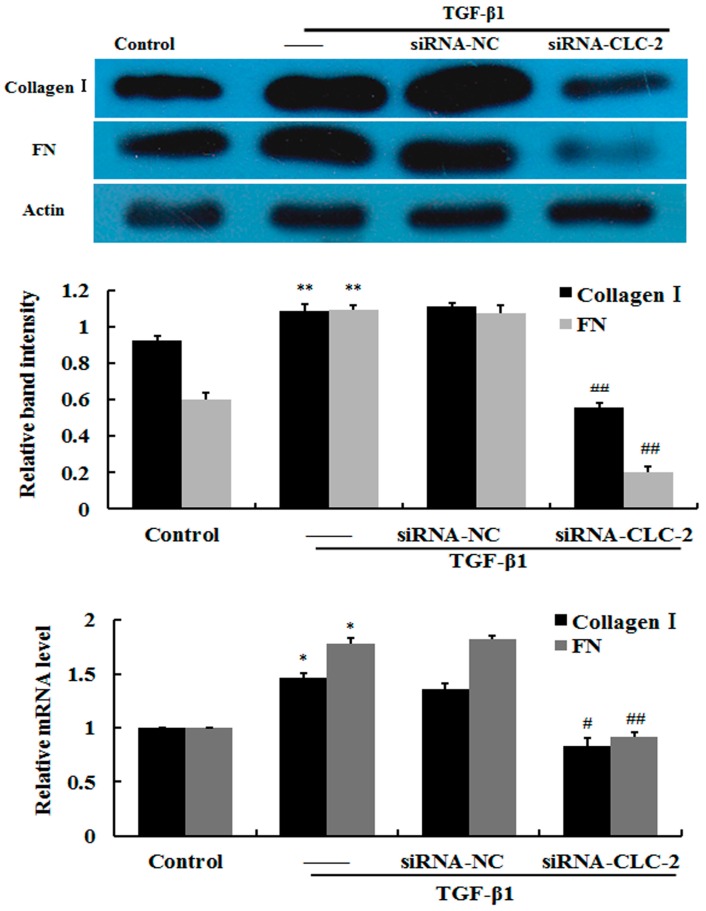
*CLC-2* knockdown attenuates transforming growth factor (TGF)-β1-induced collagen I and fibronectin expression. Human conjunctival fibroblasts (HConF) treated with TGF-β1 had elevated levels of collagen I and fibronectin expression compared to untreated control cells. Cells transfected with *CLC-2* siRNA in the presence of TGF-β1 showed decreased mRNA and protein expression of collagen I and fibronectin compared to nontransfected and mutant *CLC-2* siRNA transfected cells, whereas nontransfected and mutant siRNA transfected cells had similar collagen I and fibronectin expression levels; Data are presented as the mean ± SD (*n* = 4); FN: fibronectin; siRNA-NC: mutant *CLC-2* siRNA; * *p* < 0.05, ** *p* < 0.01 *vs.* control; # *p* < 0.05, ## *p* < 0.01 *vs.* TGF-β1 in ANOVA.

To determine whether ECM accumulation plays an important role in scar formation, expression levels of matrix components in HConF were measured. Western blot and RT qPCR analysis revealed that TGF-β1-induced high levels of collagen-I and fibronectin protein and mRNA expression in HConF compared to control cells. Knockout of *CLC-2* by *CLC-2* siRNA significantly attenuated expression of collagen-I and fibronectin induced by TGF-β1. Together, these data suggest that *CLC-2* siRNA suppresses TGF-β1-induced matrix accumulation in HConF (Figure 3).

### 2.4. Effect of CLC-2 siRNA on the Migration of HConF

**Figure 4:**
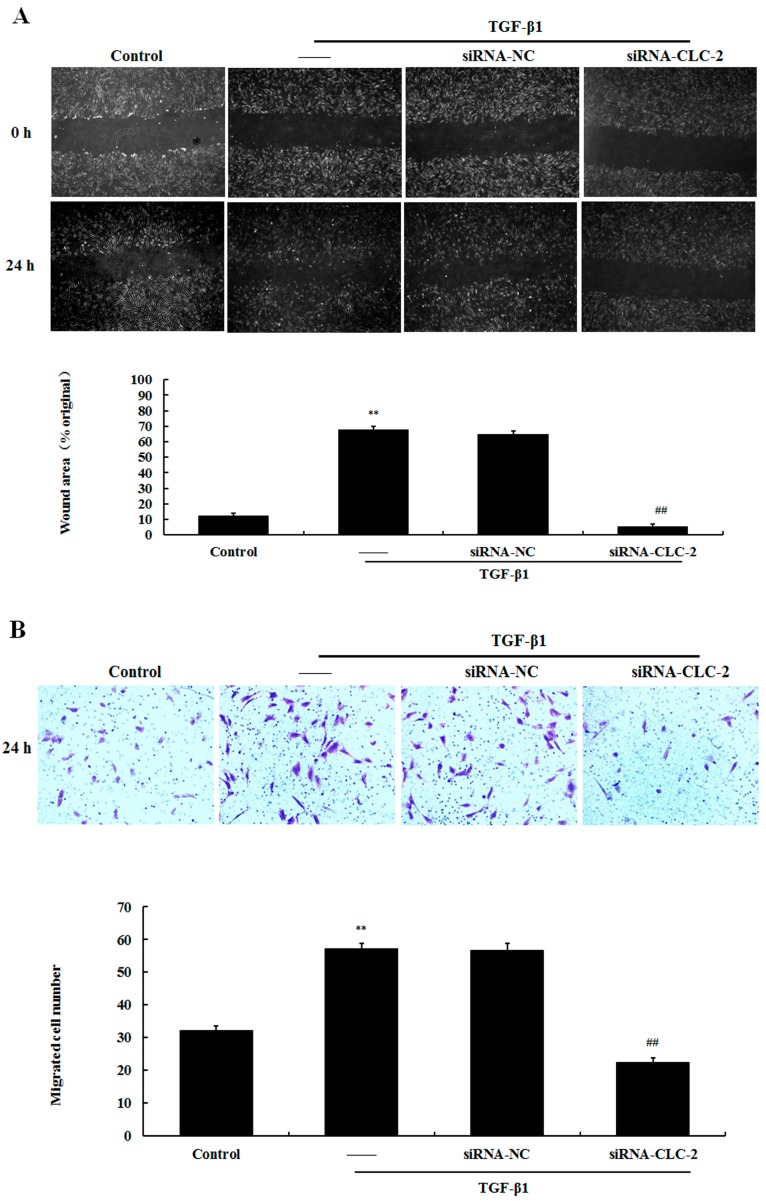
*CLC-2* knockdown inhibits transforming growth factor (TGF)-β1-induced migration in cultured Human conjunctival fibroblasts (HConF). (**A**) Confluent HconF were wounded with a sterile 200-µL pipette tipand each scratch was imaged at 0 and 24 h after wounding, migratory distance were recorded to determine the migratory ability of HconF (**B**) transwell filters were stained with crystal violet to visualize migrated cells (20× magnification), the number of HconF cells in the lower layer were recorded to determine the migratory ability of HconF. Data are presented as the mean ± SD (*n* = 4); siRNA-NC: mutant *CLC-2* siRNA; ** *p* < 0.01 *vs.* control; ## *p* < 0.01 *vs.* TGF-β1 in ANOVA.

Scratch wound and transwell migration assays were used to measure the migration capacity of HConF. In the scratch wound assay, significantly greater wound healing was observed in the TGF-β1 group compared to that in the control group (67.63 ± 2.50 *vs.* 12.41% ± 1.42%; *p* < 0.01). *CLC-2* siRNA transfection attenuated the effects of TGF-β1 on HConF migration (5.31% ± 1.44%; *p* < 0.01 *vs.* TGF-β1) (Figure 4A). Treatment with TGF-β1 resulted in significantly greater number of cells that migrated through the transwell membrane compared to that in the control group (57.26 ± 1.63 *vs.* 32.10 ± 1.24 cells; *p* < 0.01). Knockout of *CLC-2* by *CLC-2* siRNA significantly reduced the number of HConF that migrated following TGF-β1 treatment (22.34 ± 1.49 cells; *p* < 0.01 *vs.* TGF-β1) (Figure 4B).

### 2.5. CLC-2 Knockouts Suppresses TGF-β1-Induced Activation of PI3K/Akt Signaling Pathways in HConF

**Figure 5:**
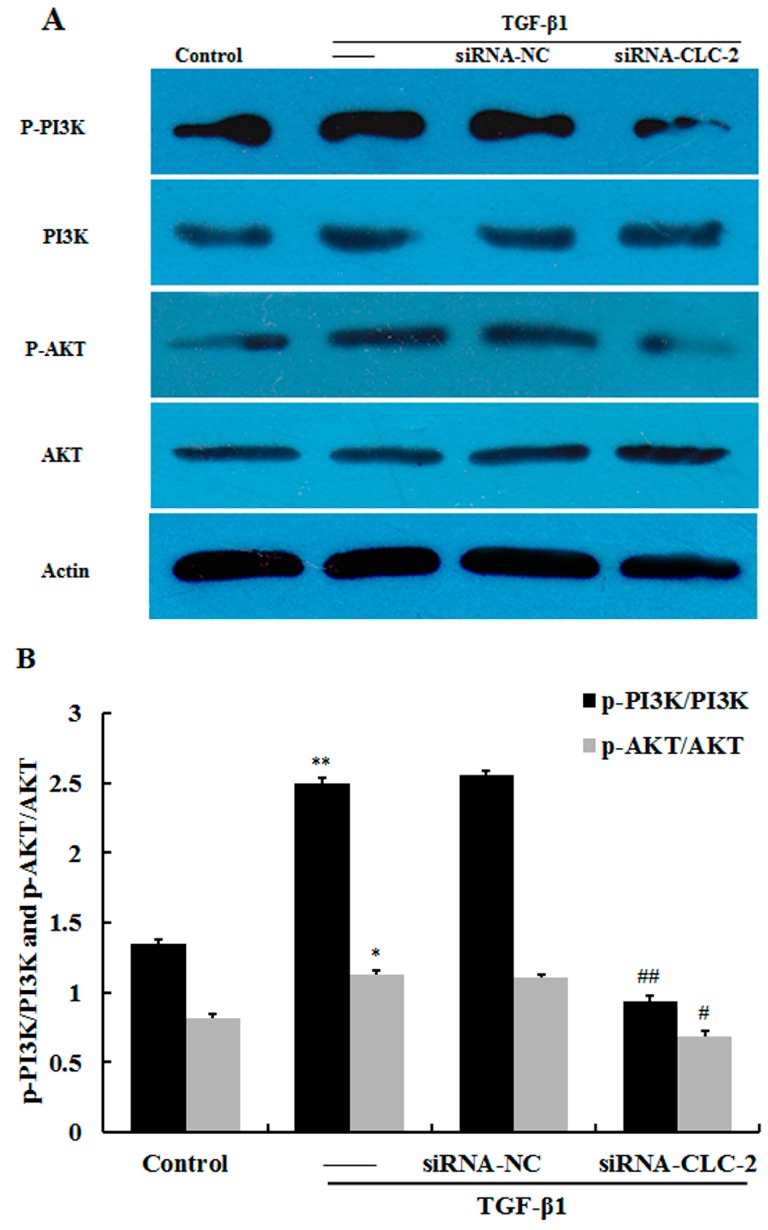
*CLC-2* knockdown attenuates TGF-β1-induced PI3K and Akt signaling (**A**,**B**) Human conjunctival fibroblasts (HconF) treated with transforming growth factor (TGF)-β1 exhibited elevated phosphorylation of PI3K and Akt compared to untreated control cells. Cells transfected with *CLC-2* siRNA in the presence of TGF-β1 showed decreased phosphorylation of PI3K and Akt compared to nontransfected and mutant *CLC-2* siRNA transfected cells, whereas nontransfected and mutant siRNA transfected cells showed similar phosphorylation of PI3K and Akt levels. Data are presented as the mean ± SD (*n* = 4); siRNA-NC: mutant *CLC-2* siRNA; * *p* < 0.05, ** *p* < 0.01 *vs.* control; # *p* < 0.05, ## *p* < 0.01 *vs.* TGF-β1 in ANOVA.

TGF-β1 significantly increased the phosphorylation of PI3K and Akt as determined by western blot (*p* < 0.01 and *p* < 0.05 respectively *vs.* control) (Figure 5A,B). Knockout of *CLC-2* decreased the levels of p-PI3K and p-Akt following TGF-β1 treatment (*p* < 0.01 and *p* < 0.05 respectively *vs.* TGF-β1).

## 3. Discussion

In the present study, we show that TGF-β1-induced the migration transition to myofibroblasts and ECM synthesis (collagen I and fibronectin) of HconF. *CLC-2* siRNA treatment reversed these effects. We further found that *CLC-2* siRNA treatment in the presence of TGF-β1 inhibited phosphorylation of PI3K and Akt in HConF. The results of this study suggest another potential strategy for resistance to scar ring after glaucoma. The healing process after glaucoma filtration is the main determinant of surgical failure; therefore, the ability to control wound healing could improve the success of glaucoma filtration surgery in patients with glaucoma. After glaucoma filtration surgery, quiescent fibroblasts in the surrounding matrix are activated and transform into myofibroblasts; these fibroblasts proliferate and migrate into the wound site, and remodeling of the new matrix result in scar formation. The transdifferentiation of fibroblasts to myofibroblasts is a crucial step in wound healing and scarring, and is required for subsequent tissue remodeling [15]. Myofibroblasts exert increased contractile activity, which is associated with the de novo expression of α-smooth muscle actin (α-SMA) [16]. Increased amounts of α-SMA are incorporated into actin stress fibers as part of the contractile apparatus [16,17] to ensure sufficient wound healing [18]. Moreover, myofibroblasts represent an “activated” fibroblast phenotype with increased synthesis of ECM proteins [7,19]. ECM proteins (mainly collagen type I and fibronectin) have been found to play an important role in the pathogenesis of scar formation [20].

TGF-β affects conjunctival fibroblasts in the later wound healing phases, and its effects include transdifferentiation of fibroblasts into myofibroblasts [6,21] and induction of deposition of ECM [7]. Therefore, TGF-β was applied in the present study to induce a series of responses similar to wound healing. As expected, TGF-β1 was found to significantly increase migration, and transition of fibroblasts to myofibroblasts characterized by α-SMA expression, was further supported by a significant increase in expression of the ECM proteins (fibronectin and collagen I). TGF-β1 stimulation was also found to increase levels of *CLC-2* mRNA transcripts and protein expression in a time-dependent manner.

Most studies on modification of healing after filtering surgery by gene transfer methods have concentrated on inhibition of the proliferation of conjunctival fibroblasts. However, conjunctival fibroblasts treated using antiproliferative approaches remain capable of migration and consequently may contribute to scar formation [22]. Therefore, prevention of ECM synthesis, differentiation, and migration of conjunctival fibroblasts by gene transfer is a new research area for control of wound healing after glaucoma filtration.

Ion channels are formed by membrane proteins to control ion flux across plasma membranes and to efficiently regulate global cell volume. By creating local osmotic gradients, ion channels facilitate the swelling or shrinkage of cellular processes [23]. Volume-activated chloride channels (VACCs) are activated by a volume-activated Cl^−^ current when stimulated by osmotic cell swelling. The almost ubiquitous CLC-2 protein, one of the VACCs members activated by membrane hyperpolarization, a low extracellular pH, and osmotic cell swelling [24], is considered to contribute to regulation of the volume-activated chloride current [25]. In this study, we confirmed that CLC-2 plays a critical role in transdifferentiation, ECM synthesis, and migration of HConF through introduction of siRNA targeting *CLC-2* that was designed to mimic the inhibition of *CLC-2* in the presence of TGF-β1. Importantly, consistent with our hypothesis, suppression of *CLC-2* inhibited the migration, transition, and ECM synthesis of HConF. Similar to our study, *ClC-3* knockdown in the presence of TGF-β1 in human corneal keratocytes and human fetal lung fibroblasts was previously shown to reduce α-SMA protein expression, whereas α-SMA protein expression was significantly increased by *ClC-3* overexpression in the absence of TGF-β1; these findings suggest that ClC-3 is involved in the transition of fibroblasts into myofibroblasts [13]. Shuai [26] found that knockdown of *ClC-3* by transfection of *ClC-3* siRNA inhibited cell proliferation and migration in osteosarcoma cells. This work demonstrated that ClC-3 is involved in the proliferation and migration of osteosarcoma cells. Previous studies [27,28,29] showed that expression of chloride intracellular channel 4 (CLIC4), one of nine ClC family members regulating intracellular pH and cell volume, was up-regulated during TGF-β1-induced transition of fibroblasts to myofibroblasts. Moreover, inhibition of *CLIC4* significantly reduced TGF-β1-induced fibroblast-to-myofibroblast transdifferentiation characterized by α-SMA expression [29]. Downregulation of *CLIC4* also reduced TGF-β1-induced expression of ECM components in primary fibroblasts [29]. Additionally, Fuqiang *et al.* [14] found that knockout of *CLC-2* gene expression by RNAi inhibited high glucose induced migration of rat keratinocytes. The present study provides the first report on the involvement of CLC-2 channels in TGF-β1-associated fibroblast differentiation, migration, and ECM synthesis in HConF. Moreover, from these findings, we can speculate that agents that inhibit CLC-2 could alleviate the expression of factors related to myofibroblast differentiation, migration, and ECM synthesis to sequentially inhibit postoperative scarring after glaucoma surgery.

To further explore how CLC-2 participates in differentiation of conjunctival fibroblasts, migration, and ECM synthesis, we observed the effect of *CLC-2* knockouts on phosphorylation of PI3K and Akt. The PI3K/Akt pathway has been shown to play key roles in the physiology and pathophysiology of many cell types [30,31,32,33]. The key enzyme of the pathway, PI3K, converts phosphatidylinositol 4,5-biphosphate into phosphatidylinositol 3,4,5-triphosphate, which binds both Akt and 3-phosphoinositide-dependent protein kinase 1 (PDK1), allowing PDK1 to phosphorylate Akt [33,34]. The primary direct downstream target protein of PI3K is Akt. Activation of Akt causes a cascade of responses of downstream targets that regulate cellular functions. Many studies have shown that the PI3K/Akt pathway plays a key role in regulating cell proliferation and migration [33]. The ClC-3 chloride channel modulates proliferation and migration of osteosarcoma cells via the PI3K/Akt signaling pathway [26]. Likewise, studies have found that inhibition of *CLC-2* expression by RNAi or chloride channel blockers can attenuate cell proliferation and migration via the PI3K/Akt signaling pathway [14,35]. It has been reported that activation of the PI3K/Akt signaling pathway can induce the expression of ECM molecules in many cell types [36,37]. In this study, we found that phosphorylation of PI3K and Akt are inhibited by *CLC-2* siRNA treatment in HconF. Importantly, these results demonstrate that the PI3K/Akt pathway is involved in CLC-2 mediated differentiation, migration, and ECM synthesis in conjunctival fibroblasts. However, the molecular mechanisms underlying the regulation of cell proliferation, differentiation, migration, and ECM protein synthesis via the PI3K/Akt pathway are still unknown. Mammalian target of rapamycin (mTOR) is a downstream effector of Akt and is a major node of control for protein translation and ribosome biogenesis. Several reports have shown that mTOR controls mRNA translation of eukaryotic cells by two mechanisms: (1) through phosphorylation of eukaryotic translation initiation factor 4E-binding protein 1 (4EBP1), a protein that prevents translation when its non-phosphorylated form interacts with eIF4E (eukaryotic translation initiation factor 4E); and (2) through phosphorylation of RPS6 by RPS6 kinase (S6K1), which also promotes protein synthesis and cell proliferation [38,39] Previous studies showed that stimulation of mTORC1 by l-leucine boosted cell proliferation, protein synthesis, and development in zebrafish models of Roberts syndrome (RBS) in which mTOR signaling was strongly downregulated [40,41]. From these findings, we can speculate that cell proliferation, differentiation, migration, and ECM protein synthesis were regulated by the PI3K/Akt/mTOR pathway via control of protein translation and ribosome biogenesis through phosphorylation of 4EBP1 and RPS6. This speculation needs to be confirmed in future studies.

## 4. Experimental Section

### 4.1. Cell Culture

HConF were obtained from ScienCell Research Laboratories, where they were isolated from human conjunctiva (ScienCell Research Laboratories, San Diego, CA, USA). HConF were characterized by their spindle morphology and immunoreactivitywith antibodies to fibronectin. Cells were maintained and cultured in fibroblast medium (FM) (ScienCell Research Laboratories) containing fibroblast growth supplement (undisclosed formulation), 2% fetal bovine serum (FBS), 100 U/mL penicillin, and 100 µg/mL streptomycin at 37 °C in a humidified incubator with 5% CO_2_.

### 4.2. Transfection of HConF with siRNA

The sequence of the stealth siRNA duplex oligoribonucleotides against the human *CLC-2* gene was 5′-UCCUCAUGAGGAAACGCCUGCUCUU-3′, and the corresponding complementary strand is 5′-AAGAGCAGGCGUUUCCUCAUGAGGA-3′. The negative control consisted of a nonsilencing oligonucleotide sequence (nonsilencing siRNA) that does not recognize any known homology to mammalian genes. To examine oligonucleotide uptake by HConF, oligonucleotides were labeled with Alexa Fluor 488 (Invitrogen Life Technologies, Inc., Carlsbad, CA, USA). According to the manufacturer’s instructions, the *CLC-2* siRNA and Lipofectamine 2000 were diluted with Opti-MEMI (Invitrogen Life Technologies, Inc.). To form transfection complexes, the diluted *CLC-2* siRNA and Lipofectamine 2000 were mixed and combined for 20 min at room temperature. Then the transfection complexes were added to the cells. After 4 h of incubation at 37 °C, the transfection mixture was removed and the cells were further incubated in normal growth conditions for 24 h before experiments.

### 4.3. Reverse Transcription Quantitative Polymerase Chain Reaction (RT qPCR)

Total RNA was extracted from HConF using RNAiso Plus (Takara Bio Inc., Shiga, Japan), according to the manufacturer’s protocol. Single stranded cDNA templates were prepared from 500 ng of total RNA using the RT for PCR Kit (Takara Bio Inc., Shiga, Japan), according to the manufacturer’s instructions. Specific cDNA sequences were amplified by polymerase chain reaction (PCR) using the following primers (Shanghai R&S Biotechnology Co., Ltd., Shanghai, China): *CLC-2*, forward 5′-CCCTGGTCATCTTCATTCTCA-3′, reverse 5′-TAGGTGCTGCTGTCCGTATG-3′; *α-SMA*, forward 5′-ATGGTGGGAATGGGACAAAA-3′, reverse 5′-CGTGAGCAGGGTGGGATG-3′; *collagen I*, forward 5′-TCCTCTTTAGCACCCTTTCG-3′, reverse 5′-GGACCAGCAACACCATCTG-3′; fibronectin, forward 5′-CCAGCAGAGGCATAAGGTTC-3′, reverse 5′-CACTCATCTCCAACGGCATA-3′; and *GAPDH*, forward 5′-CAGGAGGCATTGCTGATGAT-3′, and reverse 5′-CAGGAGGCATTGCTGATGAT-3′. PCR amplification from cDNA was performed using a LightCycler 480 System (Roche Diagnostics, Basel, Switzerland) in a final reaction volume of 20 μL containing 2× SYBR Green mix (10 μL; Toyobo Co., Ltd., Tokyo, Japan), 1 μL of primer mix, 1 μL of template DNA, and 8 μL of diethylpyrocarbonate (DEPC)-treated water. The following cycling conditions were used: initial denaturation at 95 °C for 30 s; 40 cycles of denaturation at 95 °C for 15 s, annealing at 59 °C for 20 s, and elongation at 72 °C for 20 s; and a final extension at 72 °C for 10 min. The mRNA expression were normalized to *GAPDH* mRNA expression and was calculated using the following equation: Fold change = 2^−ΔΔ*C*t^ [37].

### 4.4. Western Blot Analysis

Cells were washed with pre cooled PBS three times and lysed in RIPA buffer (50 mM Tris (pH 7.4), 150 mM NaCl, 1% Triton X-100, 1% sodium deoxycholate, 0.1% SDS) containing a protease inhibitor (sodium orthovanadate, sodium fluoride, EDTA, leupeptin, and 1 mM PMSF) for approximately 30 min on ice. Then, the cells were scraped gently with a rubber policeman and centrifuged at 1500× *g* for 12 min at 4 °C. Protein concentrations were measured using the bicinchoninic method with the Enhanced BCA Protein Assay Kit (Beyotime, Shanghai, China).

Protein (20 μg per sample) was separated by 6%–15% sodium dodecyl sulfate-polyacrylamide gel electrophoresis (SDS-PAGE) and transferred onto polyvinylidene fluoride (PVDF) membranes. Then, the membranes were blocked with 5% nonfat dry milk in Tris buffered saline with 0.1% Tween (TBST) for 1 h at 37 °C and incubated overnight at 4 °C in TBST with rabbit polyclonal anti-CLC-2 (1:3000 dilution; Abcam, Cambridge, UK; Catalog No. ab154798), rabbit monoclonal anti-α-SMA (1:10,000 dilution; Abcam, Catalog No. ab124964), mouse monoclonal anti-collagen I (1:2500 dilution; Abcam, Catalog No. ab88147), rabbit polyclonal anti-fibronectin (1:1500 dilution; Abcam, Catalog No. ab2375), and/or mouse monoclonal anti β actin (1:1000 dilution; Beyotime Institute of Biotechnology; Catalog No. AF0003) antibodies. Following two washes with TBST, the membrane was incubated with horseradish peroxidase conjugated goat anti rabbit IgG secondary antibody (1:5000 dilution; Catalog No. A0208; Beyotime Institute of Biotechnology) or goat anti mouse IgG secondary antibody (1:5000 dilution; Catalog No. A0129; Beyotime Institute of Biotechnology) for 1 h at room temperature and washed 3 times with TBST. Enhanced chemiluminescence western blotting reagents (Beyotime, Shanghai, China) were added onto the membranes for labeling of the secondary antibody. Then, the membranes were exposed to Kodak X-ray film (Eastman Kodak Company, Rochester, NY, USA) to visualize the results. The results on X-ray film were scanned and analyzed using Image J software.

### 4.5. Scratch Wound Assay

HConF were counted, seeded into 6-well plates (5 × 10^5^ cells per well), and incubated for 24 h. The culture medium was replaced by fresh medium containing 4 μg/mL mitomycin C (Hisun Pharmaceutical, Taizhou, China), and the cells were incubated for another 2 h. A line was scratched into confluent cell monolayers with a sterile 200-µL pipette tip, and the cells were washed 3 times with PBS. The scratch wound was allowed to heal for 24 h. Micrographs of each sample were captured at 0 and 24 h and the width of the scratch wound at both time points was measured using Image J software, version 1.5 (produced by Java2HTML) to evaluate the migration capacity of HConF.

### 4.6. Transwell Migration Assay

Transwell plates (pore size, 8 μm; Costar, NY, USA) were used in the transwell migration assay. The chambers were inserted into a 24-well plate. HconF (1 × 10^5^) were suspended in 200 μL of Dulbecco’s modified Eagle medium (DMEM), and seeded into the upper chamber. Our preliminary experiments showed that a FBS concentration of 20% is optimal for observing cell migration (Figure S2). Therefore, DMEM with 20% FBS was added to the lower chamber of each well, and the cells were incubated for 24 h. The medium was removed, and non-migrated cells in the upper chamber were gently removed using a cotton swab. The migrated cells in the lower chamber were fixed with para-formaldehyde (4%) for 30 min, and then stained with crystal violet for 10 min. Micrographs of each sample were captured at a magnification of 20× and cells in 6 different fields were counted.

### 4.7. Data Analyses

Data were expressed as the mean ± standard error (SE, number of observations) and were tested for associations using analysis of variance (ANOVA). Statistical significance was defined as *p* < 0.05. All experiments were repeated four times.

## 5. Conclusions

TGF-β1-induced the migration and transition of fibroblasts to myofibroblasts characterized by α-smooth muscle actin (α-SMA) expression, supported by increased endogenous expression of CLC-2 protein and mRNA transcripts. The synthesis of ECM (collagen I and fibronectin) in HConF was enhanced by TGF-β1. *CLC-2* siRNA treatment reduced TGF-β1-induced cell migration, transition of fibroblasts to myofibroblasts and ECM synthesis of HConF. *CLC-2* siRNA treatment in the presence of TGF-β1 inhibited phosphorylation of PI3K and Akt in HConF. These findings demonstrate that CLC-2 chloride channels play a critical role in TGF-β1-induced migration, differentiation, and ECM synthesis via the PI3K/Akt signaling pathway in human conjunctival fibroblasts.

## Supplementary Materials

Supplementary materials can be found at http://www.mdpi.com/1422-0067/17/6/910/s1.

## Abbreviations

HconF	human conjunctival fibroblast
TGF-β	transforming growth factor β
α-SMA	α-smooth muscle actin
ECM	extracellular matrix
siRNA	small interfering RNA
FBS	fetal bovine serum
DMEM	Dulbecco’s modified eagle medium
VACCs	volume-activated chloride channels
PDK1	3-phosphoinositide-dependent protein kinase 1
RT qPCR	reverse transcription quantitative polymerase chain reaction
