# Supplementary material for: The CLC-2 Chloride Channel Modulates ECM Synthesis, Differentiation, and Migration of Human Conjunctival Fibroblasts via the PI3K/Akt Signaling Pathway
Source: Int J Mol Sci. 2016 Jun 9;17(6):910. doi: 10.3390/ijms17060910 (PMC4926444; doi:10.3390/ijms17060910)
Supplement: Supplementary file 1 [file ijms-17-00910-s001.pdf]

Supplementary Materials: The ClC-2 Chloride Channel Modulates ECM Synthesis, Differentiation, and Migration of Human Conjunctival Fibroblasts via the PI3K/Akt Signaling Pathway

Lixia Sun, Yaru Dong, Jing Zhao, Yuan Yin and Yajuan Zheng

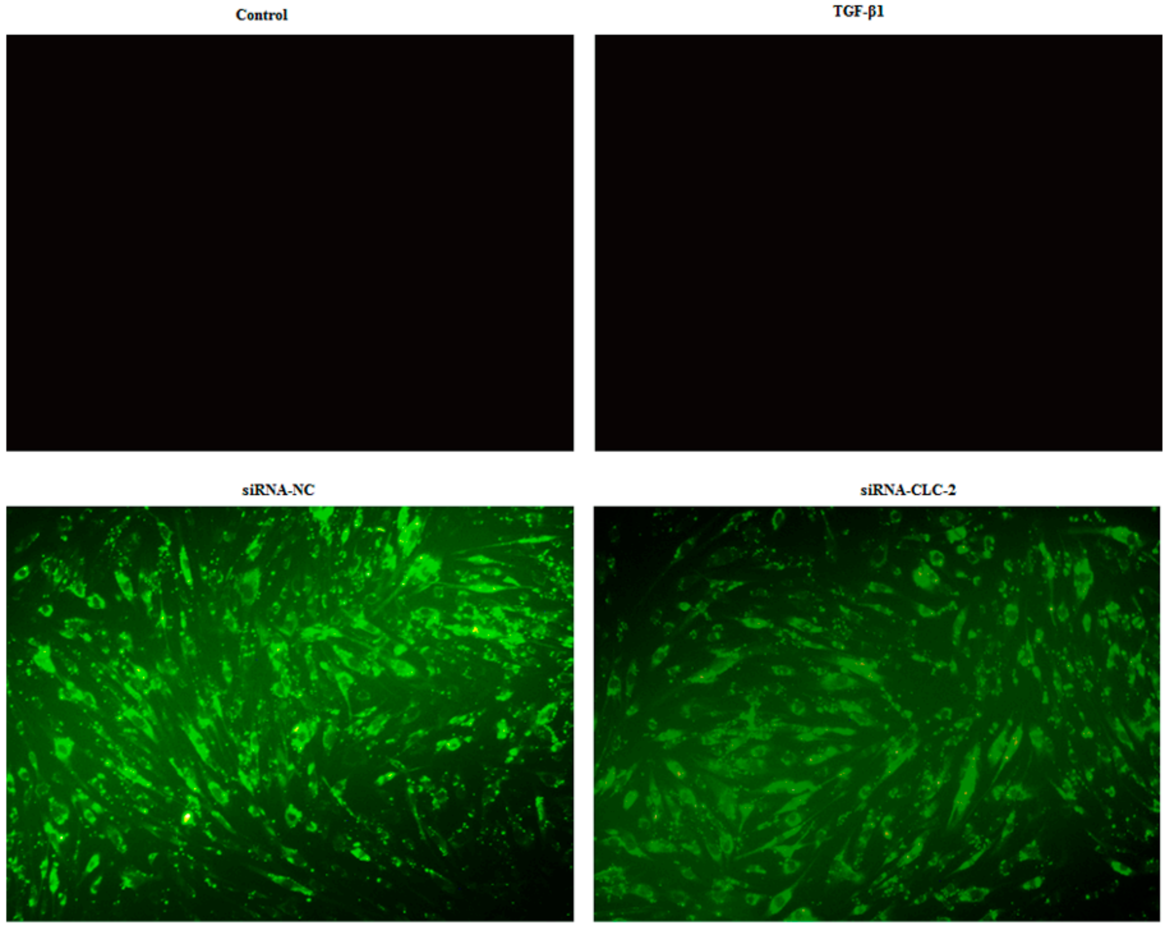

Figure S1. Fluorescence images of human conjunctival fibroblast (HconF) showing the uptake of fluorescein-labeled siRNA. Under resting conditions, cellular fluorescence was negligible, while fluorescence greatly increased in those cells treated with mutant chloride channel-2 (ClC-2) siRNA (80 nM) or CLC-2 siRNA (80 nM) which confirmed siRNA uptake by cells.

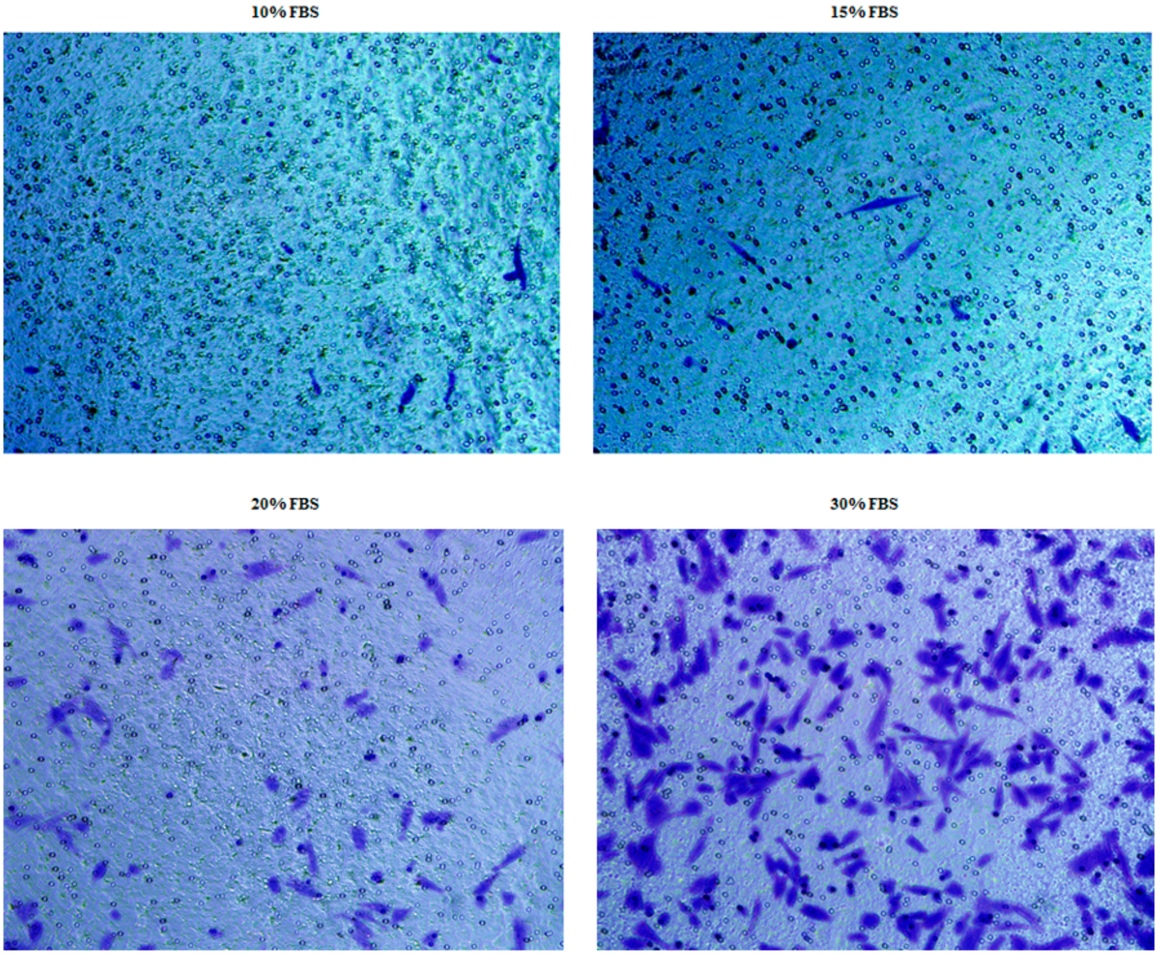

Figure S2. Optimal fetal bovine serum (FBS) concentration for observing cell migration in transwell migration assay. Dulbecco's modified eagle medium (DMEM) with different concentration of FBS was added into the lower chamber of each well and FBS was found to increase the number of cells migrated through the transwell membrane and this effect was concentration dependent. We choose 20% FBS as the optimal concentration for observing cell migration.
